# Materials and Products Development Based on a Novelty Approach to Quality and Life Cycle Assessment (QLCA)

**DOI:** 10.3390/ma17153859

**Published:** 2024-08-04

**Authors:** Dominika Siwiec, Andrzej Pacana

**Affiliations:** Faculty of Mechanical Engineering and Aeronautics, Rzeszow University of Technology, 35-959 Rzeszow, Poland; app@prz.edu.pl

**Keywords:** quality, LCA, Entropy, carbon footprint, production engineering, mechanical engineering

## Abstract

The development of materials and the products made from them should respond to new challenges posed by market changes and also by climate change. Therefore, the objective of this investigation was to develop a method that supports the sustainable development of materials and the products made from them based on an aggregated indicator of quality and environmental load in the life cycle (QLCA). The testing and illustration of the QLCA method included a passenger car tyre and nine prototypes. These prototypes were described using eight quality criteria: season, class, size of the load index, speed index, rolling, adhesion, and external noise. Then, customer expectations regarding the importance of the criteria and satisfaction with the indicators in the current and modified states were obtained. Based on the customer assessment, the quality indicators of the prototypes were assessed. This assessment was supported by the weighted sum model (WSM) and the entropy method. Then, life cycle assessment for the reference tyre was performed using the Ecoinvent database in the OpenLCA program. LCA indicators were modelled for other prototypes, taking into account quality changes. As a result of the verification of the method, an aggregated QLCA indicator was estimated, based on which it was possible to select the most favourable (qualitatively and environmentally) prototype out of nine. This was the P4 prototype (QLCA = 0.57). The next position in the ranking was taken by P7 (QLCA = 0.43). The QLCA method can be used to determine the direction of development of materials and products in terms of their sustainable development.

## 1. Introduction

The efficient and thoughtful use of materials is a current challenge, and research aims to address the challenges related to reducing the negative impact of materials manufacturing and use on the environment, including reducing resource scarcity while meeting functional requirements [1]. Therefore, improving the quality of materials and products in terms of sustainable development increasingly takes into account life cycle assessment (LCA) [2]. This involves taking actions to support the interpretation of the quality of materials and products in order to ensure customer satisfaction with their use [3,4]. At the same time, these activities should include aspects that limit the negative impact on the natural environment throughout their life cycles [5]. This is a complex and difficult approach; therefore, despite the research undertaken in this area, it is still an open topic.

For example, in one study [6], inventory data for the evaluation of environmental impact in LCA were concretised in the case of production processes and mechanical production. However, the authors of the study [7] focused their research on developing a framework for assessing products in terms of their improvement possibilities, including taking into account their life cycle. The framework included an integrated checklist to determine product development capabilities, including the assessment of specific environmental and quality parameters and indicators. The authors proposed a different approach in study [8], in which the research topic mainly referred to the use of artificial intelligence and machine learning to predict the impact of the life cycle of material products, including products made from them. In turn, the study [1] analysed the circularity of the product and the environment with the goal of improving material efficiency. The life cycle assessment method was used as a key method in estimating the carbon footprint of various material efficiency scenarios, which were successively compared with the circularity of the material. Studies have also been carried out where a method was proposed to assess the total impact of non-ferrous metals using LCA (for example, [9]) and inventory data related to the acquisition of these materials, as well as emissions from mining, transport, and production. In addition, the authors of [10] used life cycle assessment to determine the environmental impact of additively produced products, such as fibre and resin materials, e.g., polylactic acid or ultraviolet resin. However, in the study [11], an attempt was made to adapt the life cycle assessment method to the analysis of materials from the metal and mining industries, to ensure the consistency of decisions made about environmental loads. As a result, the system boundaries, the recycling allocations of by-products, and the method for selecting environmental impact assessment categories were established. Typical life cycle assessments were also carried out in [12], where the environmental impact of steel pallets used in forklifts and stationary trucks was assessed. The life cycle assessment included comparative analyses of environmental burdens. Research was also conducted to take into account customer expectations towards materials, products, and the life cycle assessment. Among other things, the study authors of the study [13] obtained customer requirements for car batteries to determine problems with their use. On the basis of their study, new batteries were designed and assessed for their environmental impact during their life cycle. In [14], a decision support model based on the LCA concept and eco-design was developed for use in the development of products, including processes. The study combined risk assessment for both humans and the environment, including life cycle assessment and economic viability. A decision tree and the LCA method were used for this purpose, and the case study was concerned with lithium-ion batteries.

After a review of the literature, it was concluded that research had been carried out in which an attempt had been made to assess the quality of materials and the products made from them using an assessment of their life cycle. Despite this, no research was found that developed methods supporting the prospective assessment of production solutions (prototypes) taking into account quality assessment (customer satisfaction with use) and life cycle environmental burden assessment (LCA). 

This was considered a research gap, which was filled by the proposed QLCA method, where Q represents quality (which is understood as customer satisfaction with the use of the product; it is assessed based on the so-called product quality criteria, that is, those that refer to customer expectations regarding the usability of the product), and LCA represents life cycle assessment. Therefore, the objective of the present investigation was to develop a method that supports the sustainable development of materials and the products made from them based on an aggregated indicator of quality and environmental load in the life cycle (QLCA). The method was created as a way of establishing a framework for the development of materials and products to ensure customer satisfaction with their use while limiting their negative environmental impact during their life cycle.

The developed QLCA method meets the requirements of modern design and production, where it is beneficial to have knowledge about customer expectations and the product life cycle. Designers and managers are required to make decisions according to environmental performance and customer requirements. Understanding the relationship between the quality of materials and products and their environmental impact can help improve their performance and aid in the adaptation of existing or new products to meet changes in the market, in the context of sustainable development [14,15].

## 2. Materials and Methods

This research includes the development of the QLCA method, which supports decision making regarding the development of materials and the products made from them. The idea of the method is to make decisions in the early stages of the development of materials and products so that these decisions support their sustainable development in terms of quality and environment. Therefore, the concept of the method involves an in-depth analysis of current materials and the products made from them in terms of meeting customer requirements (use) [16] and, at the same time, an assessment of the environmental burdens resulting from their life cycle (from the acquisition and extraction of materials to the end of their useful life). On this basis, hypothetical prototypes of materials and products can be planned, which are subsequently subjected to prospective quality assessment and life cycle assessment.

The QLCA method includes: (i) developing prototypes of materials and products; (ii) obtaining and processing customer requirements regarding their quality; (iii) prospective quality and life cycle assessment of design alternatives; (iv) aggregation of the results of quality assessment and life cycle environmental burden assessment; and (v) interpretation of results and determination of the direction of development of materials and products. Therefore, the offered QLCA method is based on the quality assessment index (Q) and the life cycle environmental impact assessment (LCA), which are aggregated into one coherent QLCA index. This indicator is created for use with a current material or product and its prototypes. According to this indicator, development decisions can be made to prospectively plan a prototype of a material or product that is the most advantageous in terms of quality, while at the same time environmentally friendly in terms of the life cycle (obtaining and extraction of materials, production, use, and end of life).

### 2.1. Assumptions of Method

Based on the literature review and previous research, the following main assumptions were made:

- the subject of the research includes materials and products that can be subjected to qualitative and environmental assessments during their life cycle [17];
- the environmental assessment is carried out according to one environmental load criterion, which is selected based on the type of materials or products being verified [18];
- environmental assessment is carried out according to the ISO 14040 standard [19] as a life cycle of materials and products, i.e., taking into account the phases of obtaining and extracting the materials, and their production, use and end of life. As a result, the LCA indicator is determined as environmental impact in the life cycle;
- the assessment of the quality of materials and products takes into account the voice of the customer (VoC) [20], and as a result, the Q (quality index) is determined [21];
- the quality index (Q) and the environmental index (LCA) are aggregated into one QLCA index, where the ranking created on this basis helps determine the most favourable direction for the development of materials and products in order to simultaneously meet customer expectations regarding usability and reduce the negative environmental impact in the life cycle.

The description of the process of creating the method and the synthetic method of its implementation is presented in the next part of the study.

### 2.2. Description Process of Method Development

**Figure 1:**
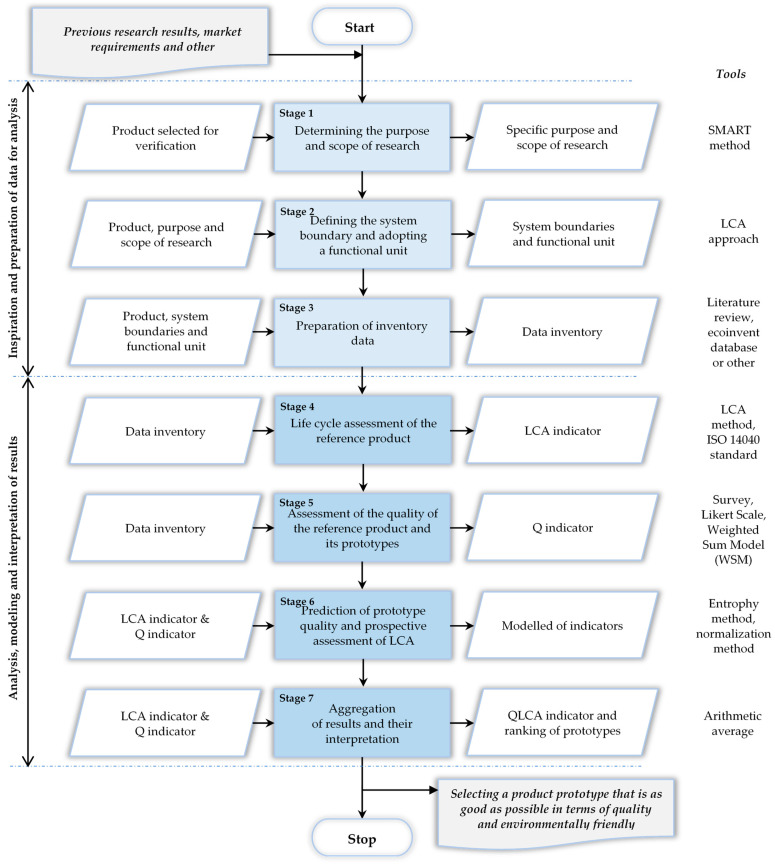
Decision making method based on prospective aggregation of the quality and life cycle assessment (LCA) metrics for material and product development. Own study.

The method surrounding the QLCA indicator was developed in seven main stages. The method was created based on the ISO 14040 standard [19], as well as according to research previously conducted by the authors of this article, e.g., [22,23]. The method implementation diagram is shown in Figure 1.

The characteristics of the main stages, along with a synthetic description of their implementation, are as follows:

1. **Determining the purpose and scope of the research.** The purpose of the research should be defined, which refers to predicting a prototype of a material or product that is the most advantageous in terms of quality (customer satisfaction with usability) [24] and, at the same time, environmentally friendly in terms of LCA [25]. The scope of research should include: (i) assessment of the quality of the materials and products and their prototypes, (ii) assessment of the environmental impact of the materials and products during their life cycle and modelling of the environmental impact of prototypes using LCA, and (iii) the integration of quality assessment with environmental assessment as part of determining the direction of material and product improvement in terms of their sustainable development. The scope should be established based on the LCA approach, i.e., from cradle-to-grave, taking into account material extraction and processing, production, use, and end of life [26].
2. **Defining the system boundary and adopting a functional unit.** A system boundary defines a set of criteria that relate to the processes, inputs, outputs, and environmental loads to be analysed. The process selected for verification may concern all or selected LCA phases, where the proposed approach assumes the entire life cycle. A system boundary is also a geographical area or time range that includes data related to a product or process [2,27]. However, the functional unit is a quantitative description of the product’s functions that corresponds to the data in the environmental load calculation process. The functional unit can be freely adopted, e.g., in international metric units. Its introduction supports the comparison of research results [28].
3. **Preparation of inventory data.** It is necessary to develop a data set to conduct a life cycle assessment of materials and reference products, as well as to assess their quality. Such data can be obtained from databases of environmental assessment programmes and catalogues (specifications). Data should be specified separately for the environmental and quality assessment processes [29]. In the case of an environmental assessment, these data should include materials and raw materials obtained and processed, along with elements implicated in the production, use, and end-of-life processes, including energy and waste [30]. However, for qualitative evaluation, it is necessary to determine the criteria (attributes) that characterise the materials and products to be analysed. These should be the main criteria that influence customer satisfaction with the use of the product. Their number should not exceed 10 [31]. Quality criteria should be described according to parameters in the current state (reference product) and modified state (prototypes), where the number of these states should not exceed 10 [31]. The current and modified states are presented by a value, range of values, or description.
4. **Life cycle assessment of the reference product.** An assessment of the life cycle of the materials and the reference product, i.e., the current product (product offered on the market) is carried out [32]. Depending on needs, a life cycle assessment is performed for selected phases of the LCA, or the entire life cycle. It is assumed that this is a “cradle-to-grave” approach, i.e., taking into account all phases of the life cycle (obtaining and extraction of materials, production, use, and end of life) [33]. Life cycle assessment is carried out according to the ISO 14040 standard [19], where the results of the life cycle assessment concern one selected environmental load criterion that is adequate for the research problem. The result of the life cycle assessment is presented by the so-called LCA index [34].
5. **Assessment of the quality of the reference product and its prototypes.** On the basis of the quality criteria (selected at the third stage of the method), customer requirements are obtained and processed. Depending on the specificity of the product, the entity using the method may obtain expectations from the customer or customers. Expectations can be obtained in various ways, e.g., via survey or interview. A survey should include product criteria and their states. The customer assesses the importance of the criteria and, in turn, satisfaction with the criteria states. Ratings are given on a five-point Likert scale, where 1 indicates that the criterion is not very important/the criterion is not very satisfactory, and 5 indicates that the criterion is very important/the criterion is very satisfactory. The methodology of the Likert scale is shown in [35]. An arithmetic mean is calculated from the customer ratings, presenting the values of the criteria weights and, in turn, the values of customer satisfaction with the quality of the criteria states. It should be mentioned that the method of data collection and analysis used may be influenced by many factors, e.g., the demographic profile of the respondents resulting from the specificity of the analysed product. In the case of a large research sample, the results from the survey may be processed using appropriately selected statistical methods or as part of the estimation of the arithmetic mean of the customer ratings awarded to a given criterion. The number of test customers can be determined according to the methods presented in [35,36]. Based on these values, it is possible to initially calculate the weighted quality of the criteria states. The WSM (weighted sum model) method [37] is used for this purpose, which is a simple decision support method based on the product of two arbitrary values, as shown in Formula (1) [38,39]:
  (1) $$q_{i}=w_{i}\times y_{i}$$
  where w is the value of the weight of the i-th criterion; y is the value of the quality of the state of the i-th criterion; and i = 1, 2, …10.

Current criteria and criteria states that satisfy customers are further analysed. They are determined on the basis of Q values, where satisfactory states are those that have Q values above the arithmetic mean value of all Q values assigned to the states of a given criterion. The way they are processed is presented in the next stage of the method.

6. **Prediction of prototype quality and prospective assessment of its life cycle.** Based on selected criteria states of materials or products, prototypes are offered, i.e., alternative design solutions corresponding to the quality of materials and products. The prototypes are defined by an expert team and should contain various modifications of the quality criteria states [40]. An effective comparison of different alternatives is possible if it covers no more than 10 production solutions [31]. Based on the proposed prototypes, the quality index (Q) is determined. For this purpose, a multicriteria decision support method known as the entropy method is used [41]. This method supports objective validity, which involves measuring various information and the weights of decision-making indicators through information entropy [42,43]. The entropy method is also suitable for analysing any quality criteria (measurable or unmeasurable) that cover various product alternatives. Therefore, the prototypes that are initially adopted and the corresponding quality criteria states are described by the values of the weighted quality of the criteria states (obtained from an earlier stage of the method). They are saved in the so-called decision matrix. Then, these values are normalized according to Formula (2) [44]:
  (2) $$r_{ij}=\frac{q_{ij}}{\sum_{i=1}^{m}q_{ij}}$$
  where q is the weighted quality of criteria states; m is the number of alternative design solutions; and i, j = 1, 2, …, n.

Next, the entropy value is calculated according to Formula (3) [42,43]:

(3) $$\left\{\begin{array}{c} e_{j}=-h\sum_{i=1}^{m}r_{ij}\ln r_{ij},j=1,2,\ldots ,n\\ h=\frac{1}{\ln m} \end{array}\right.$$

where m is the number of alternative design solutions; r is the normalized value of the weighted quality of the criterion state; and i, j, = 1, 2, …, n.

Later, a weighted vector is calculated, which is treated as an indicator of prototype quality, and then the O value is normalized to the next analysis, as shown in Formula (4) [41,44]:

(4) $$\left\{\begin{array}{c} O_{j}=\frac{1-e_{j}}{\sum_{j=1}^{n}\left(1-e_{j}\right)}\\ Q_{j}=\frac{O_{j}-minO}{maxO-minO} \end{array}\right.$$

where e is the entropy value, and j = 1, 2, …, n.

The values of the Q index should range from 0 to 1. On the basis of the Q index, it is possible to sort product prototypes from the most favourable to the least favourable in terms of meeting customer expectations. The maximum value of the Q index is the prototype that is most satisfactory to customers.

The expert then estimates changes in the environmental load in relation to changes in the parameters of the product quality criteria, based on their knowledge and experience. This is performed by estimating the percentage change in the potential environmental burden relative to the current (reference) product. This involves estimating how much (in an added or negative way) the value of the reference product load may change in relation to the environmental load of the offered prototype. These changes can be allocated by considering the changes in the quality attributes of the product prototypes, e.g., by taking into account the changing quality criteria of the product. This could include, for example, intuitively understanding the changes in the size and mass of the product, e.g., the amount of material used, and how much energy and waste is generated. The team of experts, based on their knowledge and experience, models the data using the OpenLCA program. Subsequently, the percentage value of the change in environmental load for the criteria of individual products is presented as a total value, which is recalculated in relation to the value of the environmental load of the reference product in its LCA. The modelled environmental load values are successively normalised according to Formula (5):

(5) $${LCA}_{j}=\frac{maxLCA-{LCA}_{j}}{maxLCA-minLCA}$$

where LCA is the environmental load in the life cycle of the product or prototype, and j = 1, 2,…, n.

Standardised environmental load values for the reference product and its prototypes in their LCA ensure their standardised analysis in the next step of the method. These values should range from 0 to 1, where the normalised maximum environmental load value is the first position in the ranking, which means that this product will be the most environmentally friendly in terms of LCA.

7. **Aggregation of results and their interpretation.** Values of the quality indicator (Q) and the environmental burden indicator (LCA) are aggregated into one quality and environmental indicator (QLCA). This is represented by Formula (6):
  (6) $${QLCA}_{j}=\frac{Q_{j}+{LCA}_{j}}{2}$$
  where Q is the quality indicator; LCA represents the environmental burden in the life cycle of the product or prototype; and j = 1, 2, …, n.

On its basis, it is possible to develop a ranking of prototypes in terms of simultaneously meeting customer requirements and reducing the negative environmental impact in LCA. The maximum value of the QLCA indicator is the most favourable prototype, which should be a reference for determining the direction of development of materials and products in terms of their sustainable development.

### 2.3. Data of Case Study

The testing and illustration showing how to use the method in practice were presented using the example of a passenger car tyre. Passenger car tyres are a key element of light vehicles and the only part of the vehicle in contact with the road [45]. The specificity of tyres includes essential components that are modified for proper use [46]. This not only affects customer satisfaction with use, but also has an impact on the natural environment throughout their life cycle. Therefore, it is essential to constantly strive to develop these products from a qualitative and environmental perspective. However, not much research has been conducted in this area [47]. Therefore, it was decided to analyse the passenger car tyre using the proposed QLCA method.

#### 2.3.1. Purpose and Scope of Research

The purpose of the present research was to determine a prototype of a passenger car tyre that would meet customer expectations regarding quality (usability) and, at the same time, remain environmentally friendly throughout its life cycle (LCA). The scope of this research included: (i) assessment of the quality of the reference passenger car tyre and its prototypes, where the quality assessment was made based on customer requirements (their satisfaction with the usability of the tyre); (ii) assessment of the environmental impact of the reference passenger car tyre during its life cycle, and modelling of the environmental impact prototypes of this tyre in terms of their life cycle; and (iii) integration of quality assessment with environmental assessment in order to set the direction for improving the car tyre in terms of its sustainable development. The scope covered LCA according to a cradle-to-grave approach, i.e., taking into account material extraction and processing, production, use, and end of life. It was assumed that automobile tyres were produced and used in Poland. The data for the analysis came from a literature review, the GREET v1.3.0.13991 model [48], and the Ecoinvent 3.10 database of the OpenLCA 2.0.0 program [49].

#### 2.3.2. System Boundary

**Figure 2:**
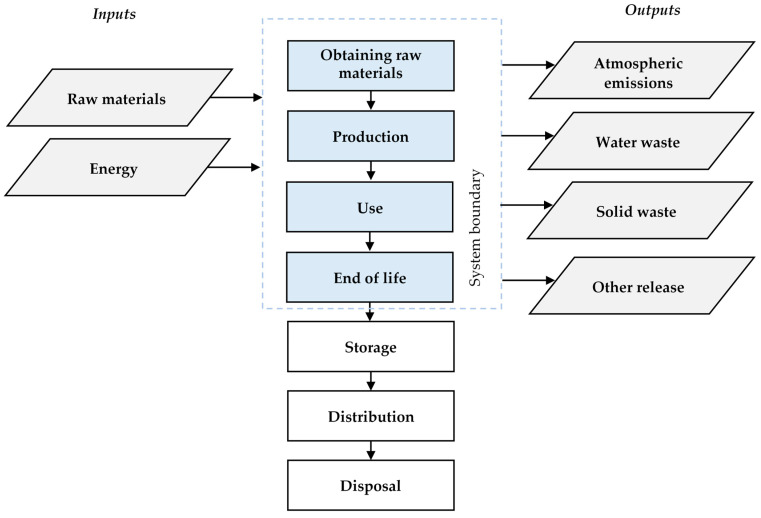
System boundaries of the passenger car tyre.

The research frontiers included the essential phases of LCA, i.e., material extraction and processing, production, use, and end of life. Energy and raw materials were taken as input elements. The outputs were ambient emissions and waste, as shown in Figure 2.

The research method included evaluating the life cycle of a passenger car tyre to assess the environmental burden of the carbon footprint (CF). The carbon footprint is a derivative of ecological footprint [50], and it is used as a key indicator in the assessment of threats related to climate change [51]. The carbon footprint is interpreted as the total amount of carbon dioxide emissions that are the result of activities caused directly and indirectly by a product. In the life cycle, it is interpreted on the basis of its phases, where the calculation unit is the carbon dioxide equivalent (eCO_2_) [52].

The choice of the carbon footprint criterion resulted from the fact that it is the key criterion in life cycle assessments of products used by society, such as the analysed passenger car tyres [53]. Additionally, reducing carbon dioxide emissions is one of the most important current challenges being undertaken in terms of sustainable development [53,54]. The choice of this criterion was also influenced by the fact that personal mobility (including passenger car use) is responsible for about 34% of the carbon footprint in households in high-income European countries [55].

It was also possible to include other environmental impact categories that may also provide some benefits given their analysis, such as water usage (to calculate the use of freshwater to produce goods and services that are consumed by customers and individuals, or produced by companies) [56]; resource depletion (as part of the analysis that allows decision-making to reduce water shortages or potential damage to ecosystems, including humans) [57]; and toxicity (toxicity to humans, which allows for estimating the potential damage of substances released into the environment) [58].

#### 2.3.3. Functional Unit

The functional unit was adopted as part of the normalisation of data for their standardised comparison. This is important because of the concepts of the research being carried out, where a reference passenger car tyre is compared with its prototypes. The functional unit included a passenger car tyre that was assumed to travel a distance of 50,000 km. A normal driving style was considered. It was assumed that the car tyre would be used for a period of 10 years. These assumptions were made on the basis of the literature on the subject, e.g., [59,60,61].

#### 2.3.4. Inventory Data

According to data from the GREET v1.3.0.13991 model, as well as data presented by the authors of previous studies [60,61,62], inventory data were adopted to assess the life cycle of a passenger car tyre. The GREET model was used to verify the components (materials and CO_2_ emissions) of a car tyre. Modelled data for life cycle assessment of a reference passenger car tyre were estimated as the arithmetic mean of the minimum and maximum values presented in [60]. The results are shown in Table 1.

Based on life cycle inventory data, a prospective evaluation of a reference passenger car tyre was performed.

As part of the evaluation of the reference quality of a passenger car tyre, it was necessary to define the quality criteria (attributes). These criteria were selected based on the catalogue of products of this type, and were the main criteria characterising this type of product, i.e., season, tyre class, size, load index, speed index, rolling resistance, wet grip, and external noise. The criteria were characterised according to sample parameters, as shown in Table 2.

The attributes of a passenger car tyre and their possible modifications were used to build product prototypes. The further process for using the proposed method is presented in the form of results from associated data processing in the next section of the study.

## 3. Results

The main results of the method, including the main stages of the method, are presented in this chapter. The results included the stages of the method, i.e., assessment of the life cycle of the reference product and its prototypes, assessment of the quality of the reference product and its prototypes, prediction of the quality of prototypes and prospective assessment of their life cycle, and aggregation of results and their interpretation.

### 3.1. Life Cycle Assessment of the Reference Product and Its Prototypes

The life cycle of a reference passenger car tyre, that is, the tyre currently on sale, was assessed. Life cycle assessment took a cradle-to-grave approach, covering the phases of material sourcing and extraction, production, use, and end of life. The ISO 14040 standard was followed, and the analysis results included the adopted criterion of environmental burden, which was represented by the carbon footprint. Based on LCA inventory data (adopted in Section 2) and data from the Ecoinvent 3.10 database data from the OpenLCA 2.0.0 program, the environmental impact of a passenger car tyre throughout its life cycle was analysed.

Carbon footprint emissions (CO_2_) for the reference tyre were estimated to be approximately 426,142,000 m^2^a [45,46,60].

**Figure 3:**
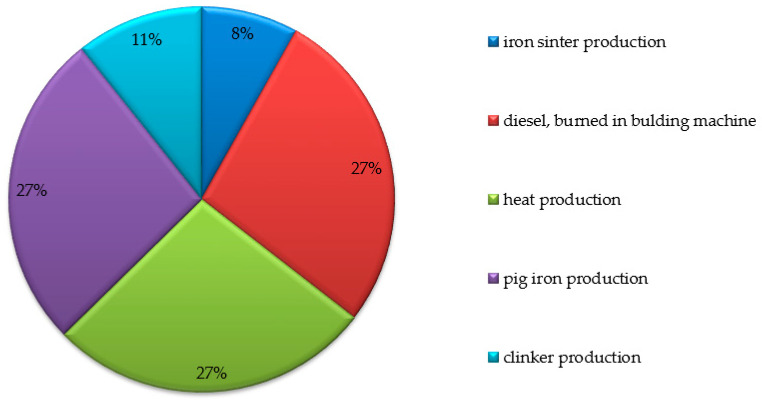
The main contributions to the results of the carbon footprint impact throughout the life cycle of a passenger car tyre.

Then, the main contributions to the results of the carbon footprint generated throughout the life cycle of a passenger car tyre were analysed. According to the OpenLCA programme models, this contribution covered the five key elements that generated the highest carbon footprint emissions in the car tyre LCA. The results obtained with the assumed assumptions are presented in Figure 3.

The greatest contribution to the formation of the carbon footprint in the LCA of a passenger car tyre is made by the use of burnt diesel oil (58,250,000 m^2^a), followed by the production of heat in an industrial hard coal furnace (57,740,000 m^2^a) [63]. Pig iron production has almost half the share of the combination of the factors previously mentioned (56,730,000 m^2^a) [60,61,64]. This is followed by clinker production (22,750,000 m^2^a) and sintered iron production (17,310,000 m^2^a) [65]. To reduce environmental burdens, including the creation of a carbon footprint, it is necessary to take improvement actions regarding these processes. Examples of improvement solutions have been presented in the work of other authors, e.g., [60,61,62].

Further processing of the results from the life cycle assessment of the reference passenger car tyre is carried out in the last stage of the method, preceded by the quality assessment presented later in the study.

### 3.2. Quality Assessment of the Reference Product and Its Prototypes

As part of the evaluation of the reference quality of a passenger car tyre and its prototypes, customer expectations were obtained regarding the importance of quality criteria and the quality of the states of these criteria. Pilot studies were carried out to cover the requirements of an individual client. Ratings were given on a five-point Likert scale. When assigning the ratings, the client was guided by economy, safety, quality, and comfort. In the case of criteria, the season and weight criteria were arbitrarily assumed to be very important, because they depend on the season and type of vehicle. Based on the customer’s requirements, in accordance with Formula (1), the weighted quality of the criteria states was calculated. Additionally, the customer satisfaction states were selected based on the weighted quality of the states of the tyre criteria. In this case, these were values ≥ 12 (defined as approximately half of the maximum value to be obtained, i.e., 25). The results are shown in Table A1. Taking into account the importance of the criteria and the quality of their states, seven main criteria necessary for further verification were selected according to the assumptions adopted. External noise turned out to be of little importance in this case, and was mostly ignored. At the same time, several criteria states did not meet the minimum assumed customer satisfaction value, so they were also omitted.

### 3.3. Prediction of Prototype Quality and Prospective Assessment of Their Life Cycle

**Figure 4:**
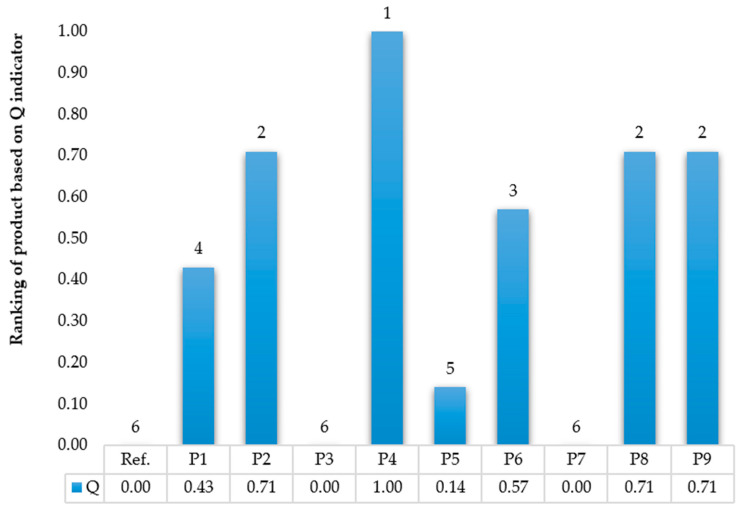
Quality indicator of planned passenger car tyre prototypes.

As part of the prediction of the quality of car tyre prototypes and the prospective assessment of their life cycle, it was initially necessary to develop a set of offered prototypes. Prototype tyres were created based on satisfactory levels of quality criteria, and 10 comprehensive possible production solutions were offered. The production solutions included various modifications of the criteria states that were sampled as part of the method test, as shown in Table A2. Then, the reference quality indices of the tyre and its prototypes was estimated. The proposed entropy method was used for this purpose. Initially, using Formula (2), the weighted qualities of the criteria states were normalised, as shown in Table A3. Then, the entropy value was calculated using Formula (3). The number of criteria for alternative design solutions was seven; therefore, the value of h was determined to be 0.51. Consequently, entropy values were calculated as shown in Table A4. Finally, the quality index of the planned passenger car tyre prototypes was determined, as shown in Figure 4.

According to the Q index, it was shown that the car tyre classified as reference was relatively undesirable in terms of quality (utility), taking into account customer expectations. The most satisfying were the P4, P2, P8, and P9 prototypes. However, this classification could vary in environmental terms.

**Figure 5:**
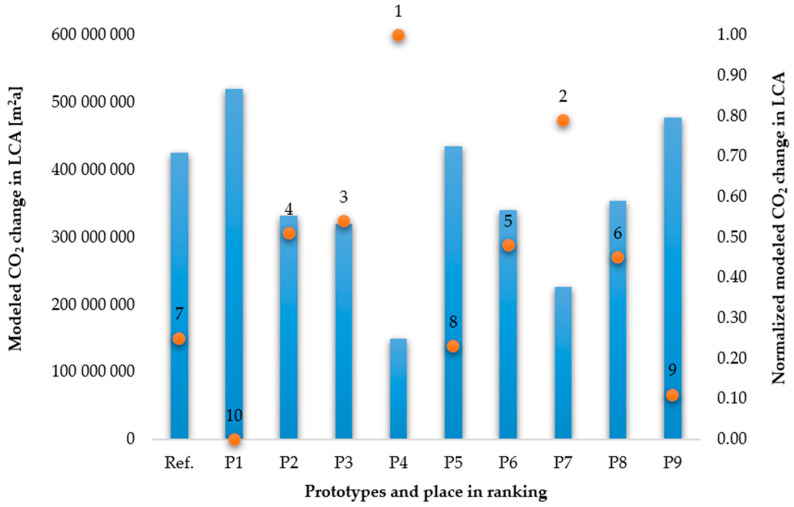
Modelled changes in the value of the carbon footprint emission factor in the life cycle of passenger car tyre prototypes.

Therefore, as part of the concept of the method, changes in environmental load were estimated based on the parameters related to changes in tyre quality criteria. This was carried out by the expert, based on his knowledge and experience, where the reference for expected changes in environmental load was the current (reference states) parameters of the tyre criteria, as presented in Table A5. According to the adopted prototypes of tyre solutions (Table A2), the total changes, in terms of the value of the total environmental load in the LCA, were modelled in relation to the changes compared to the reference tyre. These values were successively normalised according to Formula (5) in order to systematically analyse them in the next part of the study. The results are shown in Figure 5.

The results of the modelling of the environmental burden indicator with respect to the carbon footprint allowed for prospective estimation of the impact of car tyre prototypes using LCA. It was observed that the most favourable in terms of the environment was prototype 4, followed by prototype 7 and prototype 3. The least favourable were prototype 1 and prototype 9. The reference tyre was seventh in the ranking.

### 3.4. Aggregation of Results and Their Interpretation

**Figure 6:**
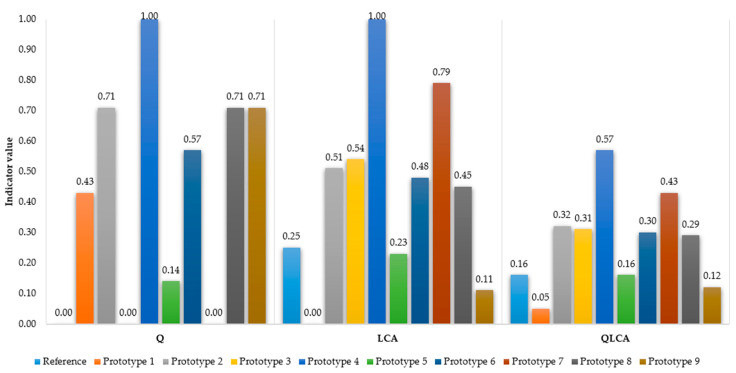
Results of QLCA assessment for passenger car tyre prototypes.

As part of the standardised analysis of passenger car tyre prototypes from a qualitative and environmental perspective, the quality indicator (Q) was aggregated with the life cycle environmental burden indicator (LCA). Formula (6) was used for this purpose. The results are shown in Figure 6.

The results of the method allowed the identification of the most advantageous production solutions in terms of meeting customer expectations regarding the quality (use) of a car tyre while at the same time limiting the negative environmental impact throughout the tyre life cycle. The most advantageous overall solution was determined to be prototype 4, which ranked first according to the QLCA index (0.57). This prototype was the most advantageous both in terms of quality (Q = 0.14) and environmental terms (LCA = 1.00). Production activities should focus on developing a car tyre that meets the assumed criteria (attributes) in terms of quality and environmental friendliness. These activities are consistent with sustainable development in that they help to meet customer expectations and, at the same time, reduce the carbon footprint of tyre products. If a production solution including prototype 4 is not possible for a company to achieve, e.g., in terms of costs, then the next prototype from the ranking should be considered; in this case, prototype 7. The final decision on the development of the product is made by the entity using the proposed method.

## 4. Discussion

The modern design of materials and the products made from them should take into account aspects of sustainable development [63,66,67]. To reduce waste, including waste of resources, it is important to work on improving the existing materials and products on the market [68]. However, it is difficult to obtain data for life cycle assessment of these materials and products, which requires additional processing and modelling, e.g., depending on qualitative or economic aspects [69]. Research in this area requires the separate acquisition and processing of different types of data, including the voice of the customer and how the life cycle assessment (LCA) was performed [70]. These analyses require a different approach, including the use of methods that support the qualitative and environmental assessment processes [71,72]. Therefore, the objective of this investigation was to develop a method that supports the sustainable development of materials and the products made from them, based on an aggregated indicator of quality and environmental load in the material or product life cycle (QLCA).

This research was supplemented with a sensitivity analysis as part of a post factum determination of the effectiveness of the methodology, including the assumptions and mutual contribution of the method’s indicators to the final QLCA indicator. A neural network was built, taking the values of the quality indicator (Q) and the life cycle environmental burden indicator (LCA) as input. The output included the values of the quality and environmental indicator (QLCA).

The neural network was created in Statistica 13.3 using machine learning techniques according to the neural network. Regression analysis was used because the verified data were quantitative. The sensitivity analysis model was developed using random sampling. The training set was divided into a training sample (70%), a test sample (15%), and a validation sample (15%). The initial value of the generator was set to 1000. After repeated testing of the available models, the model with the most favourable parameters was selected: the MLP 2-5-1 network, which had two neurones at the input, five neurones in the hidden layer, and one neurone at the output. This network had 100% training quality with the BFGS 15 learning algorithm, which has linear latent and output activation. Using the MLP 2-5-1 network, a global sensitivity analysis was performed, resulting in learning values of Q = 1.399 and LCA = 2.145, respectively. Following the authors of [73], these values were greater than 1. This also confirmed the efficiency of the method. It was observed that in the offered method, the LCA indicator had a greater impact on the final QLCA indicator than the Q indicator. The results of the sensitivity analysis in the context of their implications for the QLCA method indicate that in the proposed approach, the LCA indicator contributed a greater share than the Q indicator in the final ranking of the prototypes. This means that the results regarding the environmental burdens of the products had a greater impact on development decisions than their quality. However, this is the result of an individual case; therefore, depending on the adopted assumptions, data, customer requirements, and expert assessments, the results may be different for other cases.

The main benefits achieved by the proposed QLCA method include:

- in-depth analysis of customer requirements regarding the quality of materials and products, including their prototypes, which is aimed at predicting user satisfaction;
- ensuring a prospective life cycle assessment of materials and products, including their prototypes, based on modelling the value of environmental load in relation to the offered changes in quality criteria (attributes);
- aggregation of the quality indicator (Q), related to the quality of materials and products, with the environmental burden indicator throughout their life cycle (LCA), based on which it is possible to predict the most advantageous product alternative;
- supporting the process of improving materials and the products made from them in terms of their sustainable development in the life cycle.

However, some limitations of the method include the inability to perform a detailed analysis of the environmental loads of product prototypes during their life cycle, which, as confirmed by [2], is impossible in the early stages of product development. In the proposed method, this is performed on the basis of conventional modelling, with an indicator of the change in environmental load depending on the change in the quality attributes of the product. Furthermore, the results of the method depend on customer expectations, including the expert team involved in making decisions about the development of materials and products. Hence, the results may vary and should be individually interpreted depending on the company’s production needs. Other limitations of the method include, for example, the possibility of interpreting the environmental burden in the life cycle of a product and its prototypes according to a single assessment criterion (in this case, the carbon footprint). In the case of complex products that require consideration of a greater number of environmental burden criteria, this may be a problem. Additionally, the need to have life cycle assessment software, such as OpenLCA 2.0.0 in this case, is a limitation. Other types of software can also be used. In addition, limitations may be found in the knowledge and skills of a team of experts who participate in the assessment of qualitative and environmental aspects, including when deciding on the selection of the most advantageous prototype.

As part of future research, it is planned to extend the QLCA method to include cost aspects in order to also make development decision-making considerate of financial issues. This is an important issue because a company’s capability to improve materials and the products made from them is often dependent on budget resources.

## 5. Conclusions

The development of materials and the products made from them has been an area of interest in recent years from the point of view of their sustainable development. However, it is still an open topic. Therefore, the creation of principles and techniques for their appropriate development may contribute to improvements in the activities of manufacturing companies. Therefore, the objective of the research was to develop a method that supports the sustainable development of materials and products based on an aggregated indicator of quality and environmental load throughout the life cycle (QLCA). The method was created to set a direction for the development of materials and the products made from them that ensures customer satisfaction with their use while limiting the negative environmental impact during their life cycle.

The QLCA method was developed in seven main stages, i.e., (1) defining the purpose and scope of research, (2) defining the system boundary and adopting a functional unit, (3) developing inventory data, (4) assessing the life cycle of the reference product, (5) assessment of the quality of the reference product and its prototypes, (6) prediction of the quality of prototypes and prospective assessment of their life cycle, and (7) aggregation of results and their interpretation. The method test included a passenger car tyre and nine different prototypes (alternative production solutions). The qualitative attributes that constituted the reference for the development of tyre prototypes were season, class, size, load index, speed index, rolling resistance, and grip. The customer assessed the prototypes against quality attributes, including determining the importance of each attribute. On the basis of customer ratings, the attribute qualities of the reference tyre and its prototypes were evaluated. For this purpose, the WSM method was used to identify expected changes in quality attributes. Then, the life cycle of the reference tyre was evaluated. The criterion for the environmental burden was carbon footprint emissions. The Ecoinvent 3.10 database from OpenLCA 2.0.0 was used for life cycle assessment. Carbon footprint emissions (CO2) for the reference tyre were estimated to be approximately 426,142,000 m^2^a. Then, the quality of the tyre and its prototypes was assessed using the entropy method. Prototype 4 was found to be the most advantageous (Q = 0.14). Subsequently, the change in the value of environmental load for the prototypes was modelled in relation to the change in quality attributes (prototypes). The results were normalised, and it was shown that in this case, the most advantageous was again prototype 4 (LCA = 1.00). Finally, the indicators were aggregated into one QLCA indicator, where the most advantageous production solutions were selected, i.e., prototype 4 (first position in the ranking, QLCA = 0.57), prototype 7 (second position in the ranking, QLCA = 0.43), and prototype 2 (third position in the ranking, QLCA = 0.43). The final decision in prototype selection is dependent on production possibilities, e.g., any restrictions relating to cost-based aspects.

The results of the method were subjected to a sensitivity analysis using the Statistica 13.3 programme, where a global sensitivity analysis was performed according to a constructed neural network model. The results of the sensitivity analysis confirmed the efficiency of the method, including the significant contribution of the Q and LCA indices to the final aggregated QLCA index.

Therefore, the proposed QLCA method can be used to prospectively evaluate material prototypes and the products made from them in terms of quality (use) and life cycle environmental loads (LCA). The results of the method support the ranking of production solutions while taking into account the abovementioned quality and environmental aspects; therefore, they may be useful for establishing a sequence of improvement activities towards sustainable development. The QLCA method can be used by experts, managers and decision-makers in manufacturing companies.

## Figures and Tables

**Table 1:** Inventory data for life cycle assessment of a reference car tyre.

Phases of LCA	Input/Output	Unit	Value
Obtaining raw materials and production	synthetic rubber	kg	2.410
	natural rubber	kg	1.820
	black carbon	kg	1.870
	precipitated silica	kg	0.960
	sulphur compounds	kg	0.120
	zinc oxide	kg	0.160
	mineral and plant oils	kg	0.590
	stearic acid	kg	0.100
	rubber from recycling	kg	0.050
	steel wires	kg	1.110
	textiles	kg	0.460
	polymer-polyurethanes	kg	0.240
	ethyl acetate	kg	0.030
	substance facilitating rubber gluing-butadiene glues	kg	0.030
	antiadhesive substance-silicon	kg	0.250
	remaining solvents	kg	0.020
	water	l	45.140
	electric energy	MJ	1036.120
	others	kg	0.120
Use	furnace oil for power generation	l	6.400
	high-speed diesel for power consumption	l	0.303
End-of-life	used tyre [kg]	kg	50.000
	water [L]	l	45.140
	electric energy [MJ]	MJ	1036.120
	mineral and plant oils [kg]	kg	0.590

**Table 2:** Attributes of a reference passenger car tyre and their possible modifications.

Season	Tyre Class	Size	Load Index	Speed Index (km/h)	Rolling	Grip	External Noise (dB)
year-round	premium	205/55R16	100–800	H to 210	Class A	Class A	64–67
summer	mean	155/65R14	101–825	Q to 160	Class B	Class B	68–70
winter	economic	155/70R13	102–850	R to 170	Class C	Class C	71–75
		175/70R13	103–875	S to 180	Class D	Class D	
		185/65R15	104–900	T to 190	Class E	Class E	
		155/65R13	105–925	V to 240			
		175/70R13	106–950	W to 270			
		165/60R14	107–975	Y to 300			

Gray fields are the assumed attributes of the reference passenger car tyre.

## Data Availability

Data will be made available upon request.

## Appendix A

**Table A1:** Weighted quality of passenger car tyre criteria, taking into account customer requirements.

Criteria and Their States		Weighted Quality Criteria States
Season	All year round	25
	Summer	25
	Winter	25
Tyre class	Premium	20
	Middle	16
	Economic	12
Size	205/55R16	25
	155/65R14	25
	155/70R13	25
	175/70R13	25
	185/65R15	25
	155/65R13	25
	175/70R13	25
	165/60R14	25
Index load capacity	100–800	9
	101–825	9
	102–850	9
	103–875	12
	104–900	12
	105–925	12
	106–950	15
	107–975	15
Index speed	H to 210 km/h	12
	Q to 160 km/h	9
	R to 170 km/h	9
	S to 180 km/h	12
	T to 190 km/h	15
	V to 240 km/h	15
	W to 270 km/h	12
	Y to 300 km/h	6
Resistance rolling	Class A	10
	Class B	15
	Class C	20
	Class D	25
	Class E	10
Adhesion	Class A	12
	Class B	16
	Class C	16
	Class D	16
	Class E	8
Noise external	64–67 dB	10
	68–70 dB	8
	71–75 dB	6

Gray fields are the criteria states of a passenger car tyre that meet the customer’s expectations.

**Table A2:** Example prototypes of a passenger car tyre.

Prototypes	Season	Class	Size	Index Load Capacity	Index Speed	Resistance Rolling	Adhesion
Reference	All year round	Economic	185/65R15	106–950	V to 240 km/h	Class C	Class B
Prototype 1	All year round	Premium	205/55R16	103–875	H to 210 km/h	Class C	Class A
Prototype 2	Winter	Economic	155/70R13	104–900	S to 180 km/h	Class D	Class C
Prototype 3	All year round	Middle	175/70R13	105–925	T to 190 km/h	Class B	Class D
Prototype 4	Summer	Middle	155/65R14	101–825	Q to 160 km/h	Class B	Class B
Prototype 5	All year round	Middle	155/65R13	107–975	W to 270 km/h	Class D	Class C
Prototype 6	Winter	Premium	175/70R13	101–825	S to 180 km/h	Class B	Class C
Prototype 7	Winter	Middle	165/60R14	103–875	T to 190 km/h	Class B	Class B
Prototype 8	Summer	Premium	155/65R14	104–900	Q to 160 km/h	Class D	Class D
Prototype 9	Summer	Economic	205/55R16	105–925	H to 210 km/h	Class B	Class A

**Table A3:** Weighted quality of criteria states and normalised quality of criteria states according to planned passenger car tyre prototypes.

	Ref.		P1		P2		P3		P4		P5		P6		P7		P8		P9	
	q	r	q	r	q	r	q	r	q	r	q	r	q	r	q	r	q	r	q	r
C1	25	0.20	25	0.20	25	0.20	25	0.20	25	0.22	25	0.19	25	0.20	25	0.20	25	0.19	25	0.22
C2	12	0.09	20	0.16	12	0.09	16	0.13	16	0.14	16	0.12	20	0.16	16	0.13	20	0.15	12	0.11
C3	25	0.20	25	0.20	25	0.20	25	0.20	25	0.22	25	0.19	25	0.20	25	0.20	25	0.19	25	0.22
C4	15	0.12	12	0.10	12	0.09	12	0.10	9	0.08	15	0.11	9	0.07	12	0.10	12	0.09	12	0.11
C5	15	0.12	12	0.10	12	0.09	15	0.12	9	0.08	12	0.09	12	0.10	15	0.12	9	0.07	12	0.11
C6	20	0.16	20	0.16	25	0.20	15	0.12	15	0.13	25	0.19	15	0.12	15	0.12	25	0.19	15	0.13
C7	16	0.13	12	0.10	16	0.13	16	0.13	16	0.14	16	0.12	16	0.13	16	0.13	16	0.12	12	0.11

where: C1—season, C2—class, C3—size, C4—load index, C5—speed index, C6—rolling resistance, C7—adhesion.

**Table A4:** Entropy value of the quality of states of the criteria, assigned to planned prototypes of a passenger car tyre.

Criteria/Prototype	Ref.	P1	P2	P3	P4	P5	P6	P7	P8	P9
C1	−0.32	−0.32	−0.32	−0.32	−0.33	−0.31	−0.32	−0.32	−0.32	−0.33
C2	−0.22	−0.29	−0.22	−0.26	−0.27	−0.25	−0.30	−0.26	−0.29	−0.24
C3	−0.32	−0.32	−0.32	−0.32	−0.33	−0.31	−0.32	−0.32	−0.32	−0.33
C4	−0.25	−0.22	−0.22	−0.23	−0.20	−0.25	−0.19	−0.23	−0.22	−0.24
C5	−0.25	−0.22	−0.22	−0.26	−0.20	−0.22	−0.23	−0.26	−0.18	−0.24
C6	−0.29	−0.29	−0.32	−0.26	−0.27	−0.31	−0.26	−0.26	−0.32	−0.27
C7	−0.26	−0.22	−0.26	−0.26	−0.27	−0.25	−0.27	−0.26	−0.26	−0.24
sum	−1.91	−1.90	−1.89	−1.91	−1.88	−1.91	−1.89	−1.91	−1.89	−1.89
e_ij_	0.98	0.98	0.97	0.98	0.96	0.98	0.97	0.98	0.97	0.97

C1, season; C2, class; C3, size; C4, load index; C5, speed index; C6, rolling resistance; C7, adhesion.

**Table A5:** Predicted changes in environmental load for states of key car tyre criteria.

Key Criteria and Their Expected States		Expected Load Changes Environmental [%]
Season	All year round	reference state
	Summer	−10%
	Winter	−5%
Class	Premium	+10%
	Middle	−15%
	Economic	reference state
Size	205/55R16	+12%
	155/65R14	−15%
	155/70R13	−15%
	175/70R13	−10%
	185/65R15	reference state
	155/65R13	−15%
	175/70R13	−10%
	165/60R14	−12%
Load index	101–825	−15%
	103–875	−10%
	104–900	−7%
	105–925	−5%
	106–950	reference state
	107–975	+7%
Speed index	H to 210 km/h	−5%
	Q to 160 km/h	−15%
	S to 180 km/h	−10%
	T to 190 km/h	−10%
	V to 240 km/h	reference state
	W to 270 km/h	+10%
Rolling resistance	Class B	+5%
	Class C	reference state
	Class D	+10%
Adhesion	Class A	+15%
	Class B	reference state
	Class C	+5%
	Class D	+10%

## References

[1] Walker S., Coleman N., Hodgson P., Collins N., Brimacombe L. (2018). Evaluating the Environmental Dimension of Material Efficiency Strategies Relating to the Circular Economy. Sustainability.

[2] Proske M., Finkbeiner M. (2020). Obsolescence in LCA–Methodological Challenges and Solution Approaches. Int. J. Life Cycle Assess..

[3] Gajdzik B. (2022). Frameworks of the Maturity Model for Industry 4.0 with Assessment of Maturity Levels on the Example of the Segment of Steel Enterprises in Poland. J. Open Innov. Technol. Mark. Complex..

[4] Gawlik R., Fuxman L., Delener N., Lu V., Rivera-Solis L. (2008). Preliminary Criteria Reduction for the Application of Analytic Hierarchy Process Method. Evolution and Revolution in the Global Economy: Enhancing Innovation and Competitiveness Worldwide.

[5] Siwiec D., Pacana A. (2021). Model of Choice Photovoltaic Panels Considering Customers’ Expectations. Energies.

[6] Zhao G., Ruan D., Wang Q., Zhang X., Wang Y. (2018). Systemic Boundaries in Industrial Systems: A New Concept Defined to Improve LCA for Metallurgical and Manufacturing Systems. J. Clean. Prod..

[7] Cordella M., Sanfelix J., Alfieri F. (2018). Development of an Approach for Assessing the Reparability and Upgradability of Energy-Related Products. Procedia CIRP.

[8] Ibn-Mohammed T., Mustapha K.B., Abdulkareem M., Fuensanta A.U., Pecunia V., Dancer C.E.J. (2023). Toward Artificial Intelligence and Machine Learning-Enabled Frameworks for Improved Predictions of Lifecycle Environmental Impacts of Functional Materials and Devices. MRS Commun..

[9] Itsubo N., Yamamoto R. (1999). Application of Life Cycle Assessment to Manufacturing of Nonferrous Metals. J. Jpn. Inst. Met..

[10] Ulkir O. (2023). Energy-Consumption-Based Life Cycle Assessment of Additive-Manufactured Product with Different Types of Materials. Polymers.

[11] Santero N., Hendry J. (2016). Harmonization of LCA Methodologies for the Metal and Mining Industry. Int. J. Life Cycle Assess..

[12] Zacchei E., Tadeu A., Almeida J., Esteves M., Santos M.I., Silva S. (2022). Design of New Modular Metal Pallets: Experimental Validation and Life Cycle Analysis. Mater. Des..

[13] Adriyanti A.L., Sahroni T.R. (2023). Design Sustainability for Battery Packaging to Increase Customer Satisfaction. J. Eng..

[14] Kulatunga A.K., Karunatilake N., Weerasinghe N., Ihalawatta R.K. (2015). Sustainable Manufacturing Based Decision Support Model for Product Design and Development Process. Procedia CIRP.

[15] Malindzak D., Pacana A., Pacaiova H. (2017). An Effective Model for the Quality of Logistics and Improvement of Environmental Protection in a Cement Plant. Przem. Chem..

[16] Ostasz G., Siwiec D., Pacana A. (2022). Model to Determine the Best Modifications of Products with Consideration Customers’ Expectations. Energies.

[17] Berglund L., Breedveld L., Oksman K. (2020). Toward Eco-Efficient Production of Natural Nanofibers from Industrial Residue: Eco-Design and Quality Assessment. J. Clean. Prod..

[18] Ashby M.F. (2009). Materials and the Environment: Eco-Informed Material Choice.

[19] Finkbeiner M., Inaba A., Tan R., Christiansen K., Klüppel H.-J. (2006). The New International Standards for Life Cycle Assessment: ISO 14040 and ISO 14044. Int. J. Life Cycle Assess..

[20] Shen Y., Zhou J., Pantelous A.A., Liu Y., Zhang Z. (2022). A Voice of the Customer Real-Time Strategy: An Integrated Quality Function Deployment Approach. Comput. Ind. Eng..

[21] Neira-Rodado D., Ortíz-Barrios M., De la Hoz-Escorcia S., Paggetti C., Noffrini L., Fratea N. (2020). Smart Product Design Process through the Implementation of a Fuzzy Kano-AHP-DEMATEL-QFD Approach. Appl. Sci..

[22] Pacana A., Siwiec D. (2024). Procedure for Aggregating Indicators of Quality and Life-Cycle Assessment (LCA) in the Product-Improvement Process. Processes.

[23] Siwiec D., Pacana A. (2024). Predicting Design Solutions with Scenarios Considering the Quality of Materials and Products Based on a Life Cycle Assessment (LCA). Materials.

[24] Wang F., Li H., Liu A., Zhang X. (2015). Hybrid Customer Requirements Rating Method for Customer-Oriented Product Design Using QFD. J. Syst. Eng. Electron..

[25] Barecka M.H., Zbicinski I., Heim D. (2016). Environmental, Energy and Economic Aspects in a Zero-Emission Façade System Design. Manag. Environ. Qual. Int. J..

[26] Gao L., Wang Z., Wang Y., Peng T., Liu W., Tang R. (2023). LCA-Based Multi-Scenario Study on Steel or Aluminum Wheel Hub for Passenger Vehicles. Procedia CIRP.

[27] Chevalier J.L., Le Téno J.F. (1996). Requirements for an LCA-Based Model for the Evaluation of the Environmental Quality of Building Products. Build. Environ..

[28] Lagerstedt J., Luttropp C., Lindfors L.-G. (2003). Functional Priorities in LCA and Design for Environment. Int. J. Life Cycle Assess..

[29] Park P.-J., Tahara K., Inaba A. (2007). Product Quality-Based Eco-Efficiency Applied to Digital Cameras. J. Environ. Manag..

[30] Karaman Öztaş S. (2018). The Limitations of LCA Methodology Towards Sustainable Construction Materials. Proceedings of 3rd International Sustainable Buildings Symposium.

[31] Mu E., Pereyra-Rojas M. (2017). Practical Decision Making.

[32] Marmiroli B., Messagie M., Dotelli G., Van Mierlo J. (2018). Electricity Generation in LCA of Electric Vehicles: A Review. Appl. Sci..

[33] Grenz J., Ostermann M., Käsewieter K., Cerdas F., Marten T., Herrmann C., Tröster T. (2023). Integrating Prospective LCA in the Development of Automotive Components. Sustainability.

[34] Lund H., Mathiesen B.V., Christensen P., Schmidt J.H. (2010). Energy System Analysis of Marginal Electricity Supply in Consequential LCA. Int. J. Life Cycle Assess..

[35] Sullivan G.M., Artino A.R. (2013). Analyzing and Interpreting Data From Likert-Type Scales. J. Grad. Med. Educ..

[36] Siwiec D., Pacana A. (2021). A Pro-Environmental Method of Sample Size Determination to Predict the Quality Level of Products Considering Current Customers’ Expectations. Sustainability.

[37] Sorooshian S., Parsia Y. (2019). Modified Weighted Sum Method for Decisions with Altered Sources of Information. Math. Stat..

[38] Kaddani S., Vanderpooten D., Vanpeperstraete J.-M., Aissi H. (2017). Weighted Sum Model with Partial Preference Information: Application to Multi-Objective Optimization. Eur. J. Oper. Res..

[39] Al-Bayati I.I., Al-Zubaidy S.S. (2020). Applying the Analytical Hierarichy Process and Weighted Sum Model for Small Project Selection in Iraq. IOP Conf. Ser. Mater. Sci. Eng..

[40] Halvorsen K. (2013). Team Decision Making in the Workplace. J. Appl. Linguist. Prof. Pract..

[41] Shanmugapriya P., Selvakumari K., Kavitha S. (2024). Entropy Method of Multi-Attribute Decision-Making Problems. E3S Web Conf..

[42] Manjate E.P.A., Saadat M., Toriya H., Inagaki F., Kawamura Y. (2022). Application of Entropy Method for Estimating Factor Weights in Mining-Method Selection for Development of Novel Mining-Method Selection System. J. Sustain. Min..

[43] Qu W., Li J., Song W., Li X., Zhao Y., Dong H., Wang Y., Zhao Q., Qi Y. (2022). Entropy-Weight-Method-Based Integrated Models for Short-Term Intersection Traffic Flow Prediction. Entropy.

[44] Zhu Y., Tian D., Yan F. (2020). Effectiveness of Entropy Weight Method in Decision-Making. Math. Probl. Eng..

[45] Hooftman N., Messagie M., Joint F., Segard J.-B., Coosemans T. (2018). In-Life Range Modularity for Electric Vehicles: The Environmental Impact of a Range-Extender Trailer System. Appl. Sci..

[46] Hennequin T., van Vlimmeren L., Mostoni S., Pomilla F.R., Scotti R., Stauch C., van der Hulst M.K., Huijbregts M.A.J., van Zelm R. (2024). Environmental Impact Prediction of a New Tire Vulcanization Activator. ACS Sustain. Chem. Eng..

[47] Pacana A., Bednarova L., Liberko I., Woźny A. (2014). Effect of Selected Production Factors of the Stretch Film on Its Extensibility. Przem. Chem..

[48] Wong E.Y.C., Ho D.C.K., So S., Tsang C.-W., Chan E.M.H. (2021). Life Cycle Assessment of Electric Vehicles and Hydrogen Fuel Cell Vehicles Using the GREET Model—A Comparative Study. Sustainability.

[49] Ciroth A. (2007). ICT for Environment in Life Cycle Applications OpenLCA—A New Open Source Software for Life Cycle Assessment. Int. J. Life Cycle Assess..

[50] Kazemzadeh E., Fuinhas J.A., Salehnia N., Koengkan M., Silva N. (2023). Assessing Influential Factors for Ecological Footprints: A Complex Solution Approach. J. Clean. Prod..

[51] García A., Monsalve-Serrano J., Martinez-Boggio S., Soria Alcaide R. (2023). Carbon Footprint of Battery Electric Vehicles Considering Average and Marginal Electricity Mix. Energy.

[52] Han J., Tan Z., Chen M., Zhao L., Yang L., Chen S. (2022). Carbon Footprint Research Based on Input–Output Model—A Global Scientometric Visualization Analysis. Int. J. Environ. Res. Public Health.

[53] Shi S., Yin J. (2021). Global Research on Carbon Footprint: A Scientometric Review. Environ. Impact. Assess. Rev..

[54] Shigetomi Y., Kanemoto K., Yamamoto Y., Kondo Y. (2021). Quantifying the Carbon Footprint Reduction Potential of Lifestyle Choices in Japan. Environ. Res. Lett..

[55] Vélez A.M.A. (2023). Economic Impacts, Carbon Footprint and Rebound Effects of Car Sharing: Scenario Analysis Assessing Business-to-Consumer and Peer-to-Peer Car Sharing. Sustain. Prod. Consum..

[56] Pfister S., Boulay A.-M., Berger M., Hadjikakou M., Motoshita M., Hess T., Ridoutt B., Weinzettel J., Scherer L., Döll P. (2017). Understanding the LCA and ISO Water Footprint: A Response to Hoekstra (2016) “A Critique on the Water-Scarcity Weighted Water Footprint in LCA”. Ecol. Indic..

[57] Siwiec D., Bednárová L., Pacana A., Zawada M., Rusko M. Decision Support in the Selection of Fluorescent Penetrants for Industrial Non-Destructive Testing. PrzemysŁ Chemiczny.

[58] Hertwich E.G., Mateles S.F., Pease W.S., McKone T.E. (2001). Human Toxicity Potentials for Life-cycle Assessment and Toxics Release Inventory Risk Screening. Environ. Toxicol. Chem..

[59] Car Tire Lifespan. https://www.supaquick.com/how-long-do-tyres-last.

[60] Piotrowska K., Kruszelnicka W., Bałdowska-Witos P., Kasner R., Rudnicki J., Tomporowski A., Flizikowski J., Opielak M. (2019). Assessment of the Environmental Impact of a Car Tire throughout Its Lifecycle Using the LCA Method. Materials.

[61] Hennequin T., Huijbregts M.A.J., van Zelm R. (2023). The Influence of Consumer Behavior on the Environmental Footprint of Passenger Car Tires. J. Ind. Ecol..

[62] Katarzyna P., Izabela P., Patrycja B.-W., Weronika K., Andrzej T. (2020). LCA as a Tool for the Environmental Management of Car Tire Manufacturing. Appl. Sci..

[63] Bianco I., Panepinto D., Zanetti M. (2021). End-of-Life Tyres: Comparative Life Cycle Assessment of Treatment Scenarios. Appl. Sci..

[64] Stokłosa J., Bartnik M. (2022). Influence of Tire Pressure on the Vehicle Braking Distance. Arch. Automot. Eng. Arch. Motoryz..

[65] Dong Y., Zhao Y., Hossain M.U., He Y., Liu P. (2021). Life Cycle Assessment of Vehicle Tires: A Systematic Review. Clean. Environ. Syst..

[66] Pacana A., Siwiec D. (2021). Analysis of the Possibility of Used of the Quality Management Techniques with Non-Destructive Testing. Teh. Vjesn. Tech. Gaz..

[67] Pacana A., Siwiec D., Bednárová L., Petrovský J. (2023). Improving the Process of Product Design in a Phase of Life Cycle Assessment (LCA). Processes.

[68] Eriksson O., Carlsson Reich M., Frostell B., Björklund A., Assefa G., Sundqvist J.-O., Granath J., Baky A., Thyselius L. (2005). Municipal Solid Waste Management from a Systems Perspective. J. Clean. Prod..

[69] Turconi R., Boldrin A., Astrup T. (2013). Life Cycle Assessment (LCA) of Electricity Generation Technologies: Overview, Comparability and Limitations. Renew. Sustain. Energy Rev..

[70] Rebitzer G., Ekvall T., Frischknecht R., Hunkeler D., Norris G., Rydberg T., Schmidt W.-P., Suh S., Weidema B.P., Pennington D.W. (2004). Life Cycle Assessment. Environ. Int..

[71] Sakao T. (2007). A QFD-Centred Design Methodology for Environmentally Conscious Product Design. Int. J. Prod. Res..

[72] Hellweg S., Milà i Canals L. (2014). Emerging Approaches, Challenges and Opportunities in Life Cycle Assessment. Science.

[73] Naskath J., Sivakamasundari G., Begum A.A.S. (2023). A Study on Different Deep Learning Algorithms Used in Deep Neural Nets: MLP SOM and DBN. Wirel. Pers. Commun..

